# Prevalence and Features of Post-stroke Urinary Incontinence: A Retrospective Cohort Study

**DOI:** 10.34172/aim.2023.36

**Published:** 2023-05-01

**Authors:** Mohammad Amin Sadeghi, Sara Hemmati, Seyede Zahra Emami Razavi, Fahimeh Vahabizad, Mir Saeed Yekaninejad, Mohaddeseh Azadvari

**Affiliations:** ^1^School of Medicine, Tehran University of Medical Sciences, Tehran, Iran; ^2^School of Public Health, Tehran University of Medical Sciences, Tehran, Iran; ^3^Department of Physical Medicine and Rehabilitation, Imam Khomeini Hospital Complex, Tehran University of Medical Sciences, Tehran, Iran; ^4^Department of Neurology, Sina Hospital, Tehran University of Medical Sciences, Tehran, Iran; ^5^Department of Epidemiology and Biostatistics, School of Public Health, Tehran University of Medical Sciences, Tehran, Iran; ^6^Urology Research Center, Tehran University of medical sciences, Tehran, Iran

**Keywords:** Stroke, Urinary incontinence, Quality of life

## Abstract

**Background:**

Long-term complications of stroke, persisting for more than 6 months after the initial event, substantially reduce the quality of life (QoL) in a significant percentage of stroke survivors. In this paper, we studied the prevalence of long-term urinary incontinence (UI) in post-stroke patients. In addition, we attempted to identify patient characteristics which were associated with higher UI prevalence, higher UI severity, and less UI-associated QoL.

**Methods:**

Medical records in a tertiary referral hospital were used to contact patients who had experienced a stroke between 6 to 32 months before the study date. The patients were given the International Consultation on Incontinence Questionnaire Short Form (ICIQ-UI-SF) questionnaire for determining the presence of UI and its severity. UI-positive patients were then given the I-QOL questionnaire to determine their QoL.

**Results:**

The prevalence of UI in our study population (n=189) was 31%. Older age at the time of stroke was associated with higher UI severity (*r*=0.290) and lower QoL (*r*=-0.265). Furthermore, the presence of movement limitation was associated with higher UI prevalence (*P*<0.001, OR=3.89) and severity (*P*=0.002, *d*=1.05). Movement limitation also significantly impacted the psychological and social aspects of UI-associated QoL (*P*=0.035, *d*=-0.74). Conversely, higher body mass indices (BMIs) were associated with lower UI severity (*r*=-0.346) and higher QoL (*r*=0.281).

**Conclusion:**

In conclusion, UI continues to be prevalent in stroke survivors long after the cerebrovascular accident (CVA). As a result, these patients require continuous monitoring and UI prevention.

## Introduction

 Stroke is a major public health problem. This is not only due to its high mortality rates (especially in developing countries) but also to the high morbidity that follows in stroke survivors.^1^ Importantly, most studies on stroke morbidity only consider immediate complications following a cerebrovascular accident (CVA), whereas chronic complications which persist for a much longer time (6 months or more after the stroke) may also ensue after such events. Previous studies have reported that these chronic ramifications of stroke are even more important determinants of the quality of life (QoL) than its short-term adverse effects.^2^

 One such complication following stroke is urinary incontinence (UI). Previous studies have reported the prevalence of post-stroke UI in the first month after stroke to be between 38% to 60%.^3^ While post-stroke UI usually resolves spontaneously within 8 weeks of stroke onset (without intervention or treatment),^4^ 19% of patients may continue to have UI 6 months or later after stroke. The onset of long-term UI is reported to be associated with the extent of the ischemia or hemorrhage, the location of the lesion, and the presence of cognitive impairment and aphasia.^5-7^

 Post-stroke UI may result from a variety of direct (i.e., damaged neural pathways) and indirect (i.e., motor, cognitive, and language deficits) etiologies.^8^ UI in survivors of stroke leads to moderate or severe disability starting at 3 months post-stroke in patients under 75 years of age.^9,10^ It can affect patients’ mood, lower their QoL, and impose a significant burden on their caregivers.^11^ In addition, patients who need toilet help are more likely to have persistent bowel disorders due to poor mobility, loss of hand skills, visual and cognitive impairment, or difficulty in communicating with others. Critically, no drug has shown effectiveness against this condition so far which indicates the necessity of further studies.^12^ Nevertheless, prioritizing post-stroke complications using epidemiological studies constitutes the first step in identifying treatments for these conditions.

 This retrospective cohort study was designed to investigate the prevalence of long-term post-stroke UI. To determine the presence and classification of UI, we used the International Consultation on Incontinence Questionnaire Short Form (ICIQ-UI-SF). For studying the impact of UI on patients’ QoL, the Incontinence Quality of Life Questionnaire (I-QOL) was used. We also studied the association of potential risk factors with ICIQ-UI-SF and I-QOL scores and the prevalence of UI.

## Materials and Methods

###  Study Population

 All patients in this study were admitted to the stroke units at a tertiary referral hospital between March 2019 and March 2021. Patients were included in this study based on the following criteria: 1. Definite diagnosis of stroke (defined as a sudden, non-convulsive focal neurological failure lasting more than 24 hours) with information recorded from clinical examination and neuroradiological findings in the medical record; 2. Age over 18 years; 3. Patient’s consent for free participation in the study; 4. No history of pre-stroke neuropathic pain; and 5. Duration of at least 6 months since the stroke. Based on this design, the patients included in our study had experienced a stroke between 6 to 32 months before our study date. In addition, our patient exclusion criteria were as follows: 1. Negative neuroimaging findings; 2. Previous cerebrovascular events; 3. Subarachnoid hemorrhage; and 4. Presence of coma or severe language disorder.

 Based on the medical records, 431 patients had been admitted to our stroke units between March 2019 and March 2021. Among these, 347 patients met the inclusion criteria and were contacted. Of these patients, 205 (59%) responded, and 189 (54%) agreed to take the questionnaires and participate in the study.

###  Questionnaires

 We used the validated Persian version of the ICIQ-UI-SF to assess the presence and severity of UI.^13,14^ This questionnaire consists of 4 main questions: 1. the frequency of incontinence, 2. the amount of incontinence, 3. the effect of UI on the patient’s QoL, and 4. the circumstances in which UI manifests itself. The first three questions are on numerical scales and their sum constitutes the patient’s total score, ranging from 0 to 21. The total score is indicative of the severity of UI and the answer to the fourth question helps in classifying the type of incontinence.

 In our study, patients were considered as suffering from UI if they scored higher than 0 on the ICIQ-UI-SF. In these patients (the UI ^+^ population), the Persian version of the I-QOL questionnaire was also administered. The purpose of this questionnaire is to more accurately assess the effects of UI on patients’ QoL.^15^ The I-QOL questionnaire consists of 22 questions, each scored on a 1 (extreme) to 5 (not at all) scale. The higher the total score of the questionnaire, the higher the QoL of the patients. In additions to its total score, this questionnaire examines three subcategories of the effects of UI on QoL. These subcategories are avoidant and limiting behaviors (ALB) which consists of questions 1, 2, 3, 4, 10, 11, 13, 20; psychosocial impacts (PS) which consists of questions 5, 6, 7, 15, 16, 17, 21, and 22; and social embarrassment (SE) which consists of questions 8, 12, 14, 18, and 19. Both the total and subcategory scores are transformed to range from 0 (lowest QoL) to 100 (highest QoL) for more convenient interpretation.

###  Statistical Analysis

 In this study, we evaluated the associations between two quantitative variables using the Pearson’s correlation coefficient. Associations between a quantitative and a binary qualitative variable were tested using the *t* test. Cohen’s D was used as the measure of effect size for these analyses. Finally, associations between two qualitative variables were tested using the chi-squared test. Odds ratio (OR) was used as the measure of effect size for these analyses. In addition to these tests, Pearson’s correlation coefficients were compared using the Fischer’s Z test. In all univariate analyses using the chi-squared and *t* tests, *P* values were adjusted using the Benjamini-Hochberg correction to account for multiple testing.

 The associations which were found to be significant or close to significant in the univariate analyses were then used to construct multiple regression models using logistic regression (for onset of UI as the dependent variable) or linear regression (for ICIQ-UI-SF or I-QOL scores as the dependent variable). All models were statistically significant (*P* < 0.05) and the significance of the coefficients in these models was tested using the Wald test.

 All statistical analyses were performed using R, version 4.0.5, and alpha was set equal to 0.05.

## Results

###  Characteristics of the Study Population

 In this study, we enrolled 189 patients. The characteristics of the study population are presented in Table 1.

**Table 1:** Characteristics of the Study Population (n = 189)

**Characteristic**	**Value**
Women, n (%)	83 (44)
Hypertension, n (%)	124 (65.61)
Diabetes, n (%)	67 (35.45)
Movement limitation, n (%)	69 (36.51)
History of smoking, n (%)	60 (31.75)
Mean age at time of CVA, years (SD)	62.6 (12.63)
Mean time since CVA, months (SD)	20.05 (7.36)
Mean BMI in males (SD)	25.84 (4.33)
Mean BMI in females (SD)	27.6 (5.08)
Mean number of parities in females, n (SD)	4.95 (2.74)

BMI, body mass index, SD, standard deviation; CVA, cerebrovascular accident.

 Based on the ICIQ-UI-SF questionnaire, 58 (30.69%) members of our study population were suffering from UI. Twenty (34.48%) of these patients stated that the incontinence was present all the time, while 29 (50%) reported an urge incontinence pattern (leaks before reaching the toilet). The mean ICIQ-UI-SF score for patients with UI in our study was 10.48 (SD = 4.89).

 Furthermore, the mean total score of the I-QOL questionnaire was 69.58 (SD = 22.36). The three sub-scores of this questionnaire, i.e., ALB, SE, and PS scores were 76.83 (SD = 23.68), 66.40 (SD = 23.04), and 64.62 (SD = 25.18), respectively.

###  Determining Factors Associated with the Onset of UI

 We looked for associations between the various characteristics of our patient population and presence of UI (Table 2). Of the quantitative patient characteristics, age at the time of CVA was almost significantly different between the UI^+^ and UI^-^ populations (*P* = 0.061, *d* = 0.38). Interestingly, this difference was more pronounced in males (*P* = 0.029, *d* = 0.62) compared to females (*P* = 0.775, *d* = 0.08), where the mean age at the time of CVA was significantly higher in male patients who ended up developing UI (*M*= 67.3, *SE* = 2.31) compared to those who did not (*M* = 59.4, *SE* = 1.47).

**Table 2:** Evaluating the Associations between Patient Characteristics and Urinary Incontinence Onset

**Characteristic**	***P *** **Value**	**Effect size - Cohen’s D (** ***d*** **) for Quantitative Variables and OR for Qualitative Variable**
Age at the time of CVA (all patients)	0.061	*d* = 0.38
Age at the time of CVA (males)	0.029*	*d* = 0.62
Age at the time of CVA (females)	0.775	*d* = 0.08
Time since CVA	0.537	*d* = 0.01
BMI	0.546	*d* = 0.12
Parity (females)	0.179	*d* = 0.38
Movement limitation	< 0.001*	OR = 3.89
History of hypertension	0.088	OR = 2.00
History of smoking	0.018*	OR = 0.34
Sex	0.088	OR = 0.52
History of diabetes	0.757	OR = 0.17

BMI, body mass index, OR, odds ratio; CVA, cerebrovascular accident. ^*^P < 0.05

 Of the qualitative patient characteristics, suffering from movement limitation was significantly associated with UI (*P* < 0.001, OR = 3.89), while the association between a positive history of hypertension and UI was also close to significant (*P* = 0.088, OR = 2.00). On the other hand, a positive history of smoking was associated with a decreased prevalence of UI (*P* = 0.018, OR = 0.337), while the association between being male and a decreased prevalence of UI was also close to significant (*P* = 0.088, OR = 0.518).

 To confirm our findings and to determine the relative importance of the variables associated with UI prevalence (movement limitation, history of smoking, history of hypertension, sex, age at the time of CVA), we used them as independent variables in a logistic regression model with UI as the dependent variable (Table 3). In this model, the coefficients for movement limitation (adjusted OR [AOR] = 3.82, 95% CI [1.90, 7.89], *P* < 0.001) and history of smoking (AOR = 0.38, 95% CI [0.15, 0.93], *P* = 0.037) were significant, while the coefficient for the interaction between sex and age at the time of CVA was close to significant (AOR = 1.05, 95% CI [0.99, 1.12], *P* = 0.084). In contrast, the coefficient for history of hypertension was not significant (AOR = 1.65, 95% CI [0.77, 3.63], *P* = 0.202), possibly indicating a less substantial association with UI than movement limitation and smoking.

**Table 3:** Variables Used in the Multiple Regression Models and Their Coefficients

**Dependent Variable**	**Model Type**	**Independent Variable**	**Coefficient (for Linear Models); Adjusted OR (for Logistic Model)**	**95% CI**	***P *** **Value**
Presence of UI	Logistic regression model	Movement limitation	3.82	1.90, 7.89	< 0.001
		History of smoking	0.38	0.15, 0.93	0.037
		History of hypertension	1.65	0.77, 3.63	0.202
		Sex	0.03	0.00, 1.35	0.079
		Age at the time of CVA	0.98	0.94, 1.03	0.451
		Sex: Age at the time of CVA (interaction)	1.05	0.99, 1.12	0.084
ICIQ-UI-SF total score	Linear regression model	Movement limitation	3.275	1.42, 6.03	0.002
		Age at the time of CVA	0.079	-0.02, 0.18	0.133
		BMI	-0.239	-0.46, -0.02	0.032
I-QOL total score	Linear regression model	ICIQ-UI-SF total score	-2.115	-3.12, -1.10	< 0.001
		Age at the time of CVA	-0.133	-0.59, 0.32	0.562
		Time since CVA	-1.166	-1.80, -0.53	< 0.001

BMI, body mass index, OR, odds ratio; CVA, cerebrovascular accident.

###  Determining Factors Associated with the Severity of UI

 Next, we evaluated the associations between the patient characteristics and UI severity, as determined by the ICIQ-UI-SF questionnaire score (Table 4). Higher BMI scores were associated with lower severity of UI (*r* = -0.346). In contrast, older age at the time of CVA (*r* = 0.290) and presence of movement limitation (*P* = 0.002, *d* = 1.05) were associated with an increase in UI severity.

**Table 4:** Evaluating the Associations between Patient Characteristics and Urinary Incontinence Severity

**Characteristic**	**Qualitative Variables: ** ***P *** **Value, Cohen’s D (** ***d*** **)** **Quantitative Variables: Pearson’s Correlation Coefficient (** ***r*** **)**
Age at the time of CVA	*r* = 0.290
Time since CVA	*r* = 0.092
BMI	*r* = -0.346
Parity (females)	*r* = -0.011
Movement limitation	*P* = 0.002*, *d* = 1.05
History of hypertension	*P* = 0.954, *d* = 0.12
History of smoking	*P* = 0.954, *d* = 0.02
Sex	*P* = 0.530, *d* = 0.27
History of diabetes	*P* = 0.171, *d* = -0.51

BMI, body mass index, CVA, cerebrovascular accident; *P < 0.05

 As was the case for UI onset, we used these variables (movement limitation, age at the time of CVA, and BMI) as the independent variables in a linear regression model with the ICIQ-UI-SF total score as the dependent variable (Table 3). In this model, the coefficients for movement limitation (β = 1.148, 95% CI [1.42, 6.03], *P* = 0.002) and BMI (β = -0.239, 95% CI [-0.46, -0.02], *P* = 0.032) were significant. Therefore, presence of movement limitation and a 1 unit increase in BMI are expected to change the ICIQ-UI-SF score by 3.275 and -0.239 units, respectively. In contrast, the coefficient for age at the time of CVA was not significant (β = 0.079, 95% CI [-0.02, 0.18], *P* = 0.133). As a result, the associations between movement limitation and BMI with UI severity seem to be more substantial than that of age at the time of CVA.

###  Determining Factors Associated with the Effect of UI on QoL

**Figure 1:**
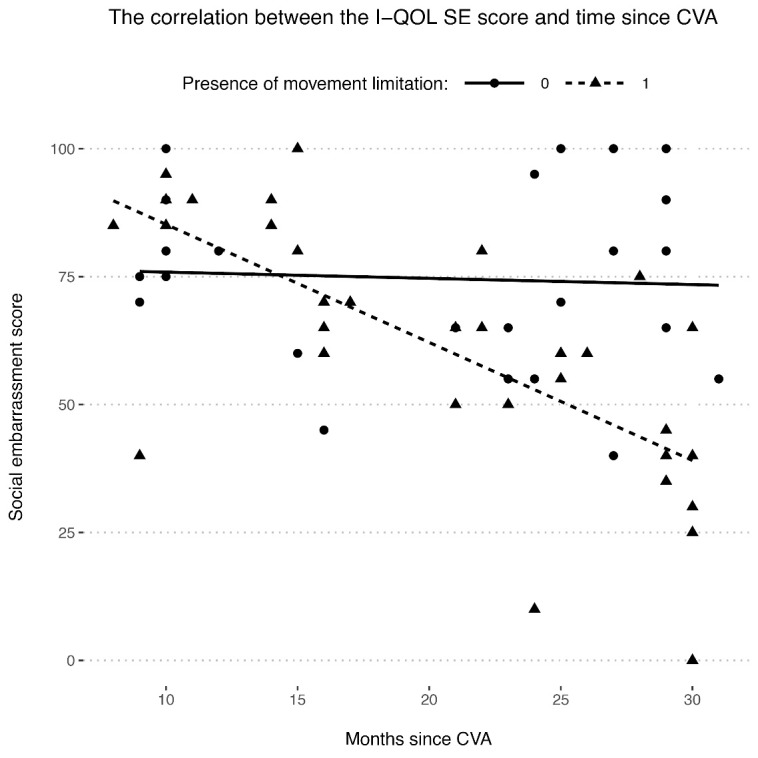
Correlation between the I-QOL social embarrassment (SE) score and time since CVA and how it is affected by the presence of movement limitation. A higher I-QOL SE score indicates a higher quality of life. There was a negative correlation between I-QOL SE score and time since CVA in the patients who suffered from movement limitation (dashed line), but the correlation was close to 0 in patients who did not have any movement limitation (solid line)

 Finally, we evaluated the factors modifying UI’s effect on QoL, as determined by the I-QOL questionnaire scores. These factors included our study population’s characteristics and their ICIQ-UI-SF scores. One observation was that time since the CVA event was negatively correlated with all scores (ALB: *r* = -0.373; SE: *r* = -0.447; PS: *r* = -0.380; total: *r* = -0.432). Interestingly, the nature of these correlations was dependent on the presence or absence of movement limitation, since the negative correlation between time since CVA and the total (w/ limitation: *r* = -0.623, w/o limitation: *r* = -0.129), SE (w/ limitation: *r* = -0.702, w/o limitation: *r* = -0.054), and PS (w/ limitation: *r* = -0.566, w/o limitation: *r* = -0.126) scores was only observed in patients with movement limitation. The difference of correlation coefficients between patients with and without movement limitation was significant or close to significant for all three scores (SE: *P* = 0.004; PS: *P*= 0.073; total score: *P* = 0.038), with the largest difference observed for the SE score (Figure 1).

 In addition to time since CVA, age at the time of CVA and UI severity (ICIQ-UI-SF score) were also negatively correlated with SE, PS, and total I-QOL scores (Table 5). In summary, the longer the time had passed from the CVA event; or the older the patient had been at the time of CVA; or the more severe the UI was, the lower would be the QoL. These correlations were especially true for the SE and PS aspects of I-QOL. In contrast, higher BMI scores were associated with higher PS scores (*r* = 0.281).

**Table 5:** Correlation Coefficients between I-QOL Questionnaire Scores, Patient Characteristics and the ICIQ-UI-SF Questionnaire Score

**Variable 1**	**Variable 2**	**Pearson’s Correlation Coefficient (** ***r*** **)**
ALB	Time since CVA	-0.373
PS	BMI	0.281
PS	Age at time of CVA	-0.285
PS	Time since CVA	-0.380
PS	ICIQ-UI-SF score	-0.669
SE	Age at time of CVA	-0.260
SE	Time since CVA	-0.447
SE	ICIQ-UI-SF score	-0.560
Total score	Age at time of CVA	-0.265
Total score	Time since CVA	-0.432
Total score	ICIQ-UI-SF score	-0.512

BMI, body mass index, CVA, cerebrovascular accident; ICIQ-UI-SF, International Consultation on Incontinence Questionnaire Short Form; ALB, avoidant and limiting behaviors; PS, psychosocial impacts; SE, social embarrassment.

 As for the qualitative variables, only movement limitation was significantly associated with QoL (Table 6). SE (*P* = 0.077, *d* = -0.65) and PS (*P* = 0.035, *d* = -0.74) scores were significantly lower in patients with movement limitation.

**Table 6:** Evaluating the Associations between Qualitative Patient Characteristics and I-QOL Scores

**Score**	**Characteristic**	***P *** **Value**	**Effect Size - Cohen’s D (** ***d*** **)**
ALB	Sex	0.613	-0.27
	History of hypertension	0.972	0.01
	History of diabetes	0.613	-0.29
	History of smoking	0.613	-0.49
	Movement limitation	0.972	-0.01
SE	Sex	0.904	-0.24
	History of hypertension	0.904	-0.08
	History of diabetes	0.904	-0.03
	History of smoking	0.904	-0.10
	Movement limitation	0.077	-0.65
PS	Sex	0.574	-0.33
	History of hypertension	0.990	0.01
	History of diabetes	0.990	0.00
	History of smoking	00.990	-0.23
	Movement limitation	0.035*	-0.74
Total score	Sex	0.592	-0.33
	History of hypertension	0.841	-0.34
	History of diabetes	0.841	-0.13
	History of smoking	0.990	-0.00
	Movement limitation	0.362	-0.48

ALB, avoidant and limiting behaviors; PS, psychosocial impacts; SE, social embarrassment.

 Finally, we built a linear regression model using the variables associated with QoL (time since CVA, ICIQ-UI-SF score, and age at time of CVA) as independent variables and total I-QOL score as the dependent variable (Table 3). Using the Wald test, the coefficients for time since CVA (β = -1.166, 95% CI [-1.80, -.53], *P* < 0.001) and ICIQ-UI-SF score (β = -2.115, 95% CI [-3.12, -1.10], *P* < 0.001) were significant. Therefore, a 1 unit increase in the ICIQ-UI-SF score or time since CVA (1 month) is expected to change the total I-QOL score by -2.115 and -1.166 units, respectively. As was the case with UI severity, the coefficient for age at the time of CVA was not significant (β = -0.133, 95% CI [-0.59, 0.32], *P* = 0.562), possibly indicating a less substantial association with UI-associated QoL in comparison to ICIQ-UI-SF score and time since CVA.

## Discussion

 UI is one of the most common long-term complications after stroke. In previous studies, the prevalence of UI six months after a stroke has been estimated to be between 19% and 34%.^11^ In our study, the prevalence of UI was 31%, which is within the range of previous reports. As mentioned before, previous studies have shown that urinary disorders decrease during the first six months following stroke. In one study, at least one urinary disorder was found in 91% of patients within one month after a stroke. In these patients, the most common complaints were related to nocturia (79.7%), frequency (78.1%), and urgency (64.1%). Six months after the stroke, the prevalence of nocturia and frequency were both 59.4%.^16^ In another study on a larger sample size of patients three months after a stroke, the prevalence of abnormal urinary symptoms was 83.6% and the most common complaints in these patients were nocturia (79.1%) and urge incontinence (17.5%).^17^ The patient population in our study had experienced stroke, on average, 20 months before our contact, and the most common type of incontinence in our patients was urge incontinence (29 patients - 50%). Previous studies have hinted at a possible etiological link between CVA and urge incontinence in which the CVA-induced brain damage may lead to an overactive detrusor muscle in the bladder.^18^

 Several features in our patient population were associated with the prevalence of UI. One interesting finding was that a positive history of smoking was associated with a lower prevalence of UI. While this finding may allude to a protective effect for smoking, another possible explanation may be selection bias, since patients suffering more severe strokes (for example, due to a positive history of smoking) were underrepresented in our study population given their higher mortality rates. In support of this alternative explanation, a case-control study conducted on 606 women found that the risk of UI was 2.5 times higher in women who had a history of smoking.^19^ Furthermore, there are several reports which associate smoking with urine leakage through sphincteric, neural, and anatomical mechanisms.^20^ In contrast to smoking, older age at the time of CVA was associated with a higher prevalence of UI which is in agreement with previous findings.^21^ Interestingly, this relationship was more substantial in males compared to females. This discrepancy may be explained by previous reports which have shown that incontinence in males tends to increase more steadily with aging whereas females have a spike in UI prevalence around menopause.^22^ Finally, as was the case with older age, presence of movement limitation was, understandably, associated with a higher prevalence of UI.

 With regards to UI severity, one interesting finding in our study was its negative correlation with BMI. This finding may be interpreted similarly to the aforementioned association between smoking and lower UI prevalence. In contrast to BMI, older age at the time of CVA and movement limitation were associated with higher UI severity. We did not find any relationship between a history of diabetes or hypertension with either increased UI prevalence or increased UI severity which is contradictory to previous reports.^23^

 The effects of UI on QoL are well documented. For example, in a previous report, which was very similar to our study in terms of the characteristics of the patient population, the reduction in QoL was more severe in patients with poor daily functionality. It was also reported that higher QoL was associated with greater independence in daily activities and mobility, higher levels of education, higher socioeconomic levels, and better social support.^24^ Indeed, in our study, the psychological and social aspects of QoL seemed to be lower in UI ^+^ patients with movement limitation. Moreover, a negative association between time since stroke and QoL was only observed in patients with movement limitation.

 Other factors that were associated with a lower QoL in our study were older age at the time of CVA and more severe UI. These findings are in line with previous reports which found that older patients who suffered from more severe UI had leakage when coughing or sneezing, urinated involuntarily more than once a day, and most of them did not seek help due to embarrassment.^25^ In contrast, we found higher BMIs to be associated with better psychosocial QoL. However, this may have been due to the negative correlation which we observed between BMI and UI severity (ICIQ-UI-SF score) in the patient population.

 In conclusion, UI continued to be prevalent in our study population long after the CVA. In these patients, older age at the time of CVA and the presence of movement limitation were associated with higher prevalence and severity of UI which in turn lead to lower QoL. The biggest impacts of movement limitation on UI-associated QoL were observed in its psychological and social aspects. In contrast, we observed higher BMIs to be associated with lower UI severity and higher UI-associated QoL. In addition, a positive history of smoking was associated with lower prevalence of UI. These findings highlight the importance of long-term complications of stroke, especially UI, in affecting the lives of stroke survivors. Furthermore, this information may be used in designing cost-effective rehabilitation and tertiary prevention programs which are targeted at the most at-risk patients.

## References

[1] Donkor ES (2018). Stroke in the 21st century: a snapshot of the burden, epidemiology, and quality of life. Stroke Res Treat.

[2] Chen Q, Cao C, Gong L, Zhang Y (2019). Health related quality of life in stroke patients and risk factors associated with patients for return to work. Medicine (Baltimore).

[3] Reding MJ, Winter SW, Hochrein SA, Simon HB, Thompson MM (1987). Urinary incontinence after unilateral hemispheric stroke: a neurologic-epidemiologic perspective. J Neurol Rehabil.

[4] Borrie MJ, Campbell AJ, Caradoc-Davies TH, Spears GF (1986). Urinary incontinence after stroke: a prospective study. Age Ageing.

[5] Gelber DA, Good DC, Laven LJ, Verhulst SJ (1993). Causes of urinary incontinence after acute hemispheric stroke. Stroke.

[6] Brittain KR, Peet SM, Castleden CM (1998). Stroke and incontinence. Stroke.

[7] Kong KH, Young S (2000). Incidence and outcome of poststroke urinary retention: a prospective study. Arch Phys Med Rehabil.

[8] Brocklehurst JC, Andrews K, Richards B, Laycock PJ (1985). Incidence and correlates of incontinence in stroke patients. J Am Geriatr Soc.

[9] Taub NA, Wolfe CD, Richardson E, Burney PG (1994). Predicting the disability of first-time stroke sufferers at 1 year. 12-month follow-up of a population-based cohort in southeast England. Stroke.

[10] Gupta A, Taly AB, Srivastava A, Thyloth M (2009). Urodynamics post stroke in patients with urinary incontinence: is there correlation between bladder type and site of lesion?. Ann Indian Acad Neurol.

[11] Nakayama H, Jørgensen HS, Pedersen PM, Raaschou HO, Olsen TS (1997). Prevalence and risk factors of incontinence after stroke. The Copenhagen Stroke Study. Stroke.

[12] Harari D, Coshall C, Rudd AG, Wolfe CD (2003). New-onset fecal incontinence after stroke: prevalence, natural history, risk factors, and impact. Stroke.

[13] Avery K, Donovan J, Peters TJ, Shaw C, Gotoh M, Abrams P (2004). ICIQ: a brief and robust measure for evaluating the symptoms and impact of urinary incontinence. Neurourol Urodyn.

[14] Hajebrahimi S, Nourizadeh D, Hamedani R, Pezeshki MZ (2012). Validity and reliability of the International Consultation on Incontinence Questionnaire-Urinary Incontinence Short Form and its correlation with urodynamic findings. Urol J.

[15] Nojomi M, Baharvand P, Kashanian M. Validation of incontinence quality of life questionnaire (I-QOL) in incontinent women. Razi J Med Sci 2009;16(63):153-61. [Persian].

[16] Akkoç Y, Yıldız N, Bardak AN, Ersöz M, Tunç H, Köklü K (2019). The course of post-stroke bladder problems and their relation with functional and mental status and quality of life: a six-month, prospective, multicenter study. Turk J Phys Med Rehabil.

[17] Williams MP, Srikanth V, Bird M, Thrift AG (2012). Urinary symptoms and natural history of urinary continence after first-ever stroke--a longitudinal population-based study. Age Ageing.

[18] Jacob L, Kostev K (2020). Urinary and fecal incontinence in stroke survivors followed in general practice: a retrospective cohort study. Ann Phys Rehabil Med.

[19] Sampselle CM, Harlow SD, Skurnick J, Brubaker L, Bondarenko I (2002). Urinary incontinence predictors and life impact in ethnically diverse perimenopausal women. Obstet Gynecol.

[20] Mobley D, Baum N (2015). Smoking: its impact on urologic health. Rev Urol.

[21] Pettersen R, Haig Y, Nakstad PH, Wyller TB (2008). Subtypes of urinary incontinence after stroke: relation to size and location of cerebrovascular damage. Age Ageing.

[22] Nitti VW (2001). The prevalence of urinary incontinence. Rev Urol.

[23] Alshenqeti AM, Almutairi RE, Keram AM (2022). Impact of urinary incontinence on quality of life among women of childbearing age in Al Madinah Al Munawara, Saudi Arabia. Cureus.

[24] Ramos-Lima MJM, Brasileiro IC, Lima TL, Braga-Neto P (2018). Quality of life after stroke: impact of clinical and sociodemographic factors. Clinics (Sao Paulo).

[25] Özcan A, Avci İ A (2021). Impact of urinary incontinence on quality of life among older adults living in a rural area of Turkey. J Gerontol Nurs.

